# NMU signaling promotes endometrial cancer cell progression by modulating adhesion signaling

**DOI:** 10.18632/oncotarget.7169

**Published:** 2016-02-03

**Authors:** Ting-Yu Lin, Fang-Ju Wu, Chia-Lin Chang, Zhongyou Li, Ching-Wei Luo

**Affiliations:** ^1^ Department of Life Sciences and Institute of Genome Sciences, National Yang-Ming University, Taipei 112, Taiwan; ^2^ Department of Obstetrics and Gynecology, Chang Gung Memorial Hospital Linkou Medical Center, Chang Gung University, Kweishan, Taoyuan 333, Taiwan

**Keywords:** neuromedin U, NMUR2, endometrial cancer, adhesion signaling, G protein-coupled receptor

## Abstract

Neuromedin U (NMU) was originally named based on its strong uterine contractile activity, but little is known regarding its signaling/functions *in utero*. We identified that NMU and one of its receptors, NMUR2, are not only present in normal uterine endometrium but also co-expressed in endometrial cancer tissues, where the *NMU* level is correlated with the malignant grades and survival of patients. Cell-based assays further confirmed that NMU signaling can promote cell motility and proliferation of endometrial cancer cells derived from grade II tumors. Activation of NMU pathway in these endometrial cancer cells is required in order to sustain expression of various adhesion molecules, such as CD44 and integrin alpha1, as well as production of their corresponding extracellular matrix ligands, hyaluronan and collagen IV; it also increased the activity of SRC and its downstream proteins RHOA and RAC1. Thus, it is concluded that NMU pathway positively controls the adhesion signaling-SRC-Rho GTPase axis in the tested endometrial cancer cells and that changes in cell motility and proliferation can occur when there is manipulation of NMU signaling in these cells either *in vitro* or *in vivo*. Intriguingly, this novel mechanism also explains how NMU signaling promotes the EGFR-driven and TGFβ receptor-driven mesenchymal transitions. Through the above axis, NMU signaling not only can promote malignancy of the tested endometrial cancer cells directly, but also helps these cells to become more sensitive to niche growth factors in their microenvironment.

## INTRODUCTION

Endometrial cancer is the most common gynecological cancer in developed countries and the second most common in developing countries [1]. With the incidence estimated in recent years [1, 2], more than 320,000 new cases worldwide will be diagnosed in 2015. Although most endometrial cancer cases are found to be of the endometrioid type and are detected at the early grade, which results in a good prognosis after surgery, approximately 15% to 20% of patients will present with a high grade of endometrioid carcinoma or other malignant subtype, such as papillary serous carcinoma or clear cell carcinoma [3]. This aggressive subset of cancers has high recurrent rates and contributes to the majority of deaths in patients [3, 4].

Although relatively few studies have been published as compared with other common cancers, several key signaling pathways have been shown to be involved in endometrial tumor development. Among these, activation of ERBB-PI3K-AKT signaling is common across all types of endometrial cancer [5]. It has been shown that the gene amplification of HER2 (ERBB2) and overexpression of EGFR (ERBB1) are respectively associated with aggressive types (papillary serous and clear cell histologies) and a poor survival rate among patients with endometrial cancer [6]. These findings suggest that this pathway may promote endometrial cancer malignancy. Activation of this pathway is inhibited by phosphatase and tensin homolog (PTEN), which is able to dephosphorylate phosphoinositide substrates. Interestingly, alterations in the *PI3K* and *PTEN* genes have been identified in 50% to 80% and 25% to 40% of all spectrums of endometrial cancer, respectively [5, 7, 8]. Also, inhibition of this pathway can impair proliferation in a diverse range of endometrial cancer cell lines *in vitro* [9, 10].

Furthermore, bioinformatics analyses of human endometrial cancer microarrays have also pointed to the fact that TGFβ pathway has a prominent role in promoting cancer aggression [11]. Various experiments on cells have suggested that this signaling is crucial for the initiation of invasion and also helps to support the survival and metastasis of endometrial cancer cells [11, 12]. In a manner similar to that of ERBB-PI3K-AKT signaling, activation of TGFβ pathway is also able to promote the epithelial-to-mesenchymal transition (EMT) through up-regulation of various transcription factors, including Twist, Snail and Slug; this further leads to loss of cell polarity and increases in cell motility and invasiveness resulting in distant metastasis.

Moreover, alternations in cell adhesion signaling have also been commonly observed during the progression of endometrial cancer. For example, CD44, a main adhesion receptor for hyaluronan, has been reported to be up-regulated in curettage and postoperative specimens of endometrial cancer [13]. Because this is usually accompanied by a significant increase in the serum levels of hyaluronan in endometrial cancer patients [14], it has been proposed that the adhesion signaling associated with CD44 is involved in the progression of endometrial cancer. Intriguingly, in addition to being directly involved in adhesion signaling, CD44 is also known to serve as a co-receptor-like molecule that is able to increase the signaling intensities of growth factor receptors, such as HER2, EGFR and TGFβ receptor; this then promotes the oncogenic activity of these receptors [15, 16]. Moreover, the situation turns out to be more complicated that, at least in fibroblasts, coupling between CD44 and EGFR can promote TGFβ signaling-driven cell proliferation [17]. Therefore, it would seem that there are synergistic effects and/or multiple interactions among ERBB, TGFβ and adhesion signaling cascades. However, such crosstalks have not yet been well explored in endometrial cancer. Also, whether there are yet other molecules that act as coordinators between these signaling pathways awaits further study.

The neuropeptide neuromedin U (NMU) was originally named based on its strong ability to cause contraction of the uterus [18]. This peptide was later found to be involved in multiplicity of functions, including regulations of feeding behavior, blood pressure, pronociception and cell development; these occur in diverse organs via binding to two different G protein-coupled receptors, designated NMUR1 and NMUR2 [19–22]. However, its functions, as well as its receptor types in the uterus, have never been characterized. In the present study, we found that NMU and NMUR2 are co-expressed in the mouse normal uterine endometrium and also in human endometrial cancer tissue samples. We also demonstrated that NMU signaling not only promotes the progression of endometrial cancers but also positively modulates the sensitivities of EGFR and TGFβ receptor via the control of adhesion signaling.

## RESULTS

### NMU and NMUR2 are co-localized in normal endometrial tissues and in endometrial cancer specimens

We first characterized the uterine receptor type for NMU. In contrast to the negligible signal obtained for NMUR1, immunohistochemical staining data indicated that NMUR2 signal can be detected in all stages within the mouse uterus tissue samples, especially the estrus stage (Supplementary Figure S1A). In the estrus stage of the mouse uterus, the transcript level of *Nmur2* was ∼300-fold higher than that of *Nmur1* (Supplementary Figure S1B). Immunohistochemical staining also indicated that the protein signals of NMUR2 and NMU are overlapped mainly in the endometrial epithelium and moderately in the myometrium (Supplementary Figure S1A and S1C), suggesting that NMU and NMUR2 may compose an autocrine and/or paracrine system in the uterus.

**Figure 1:**
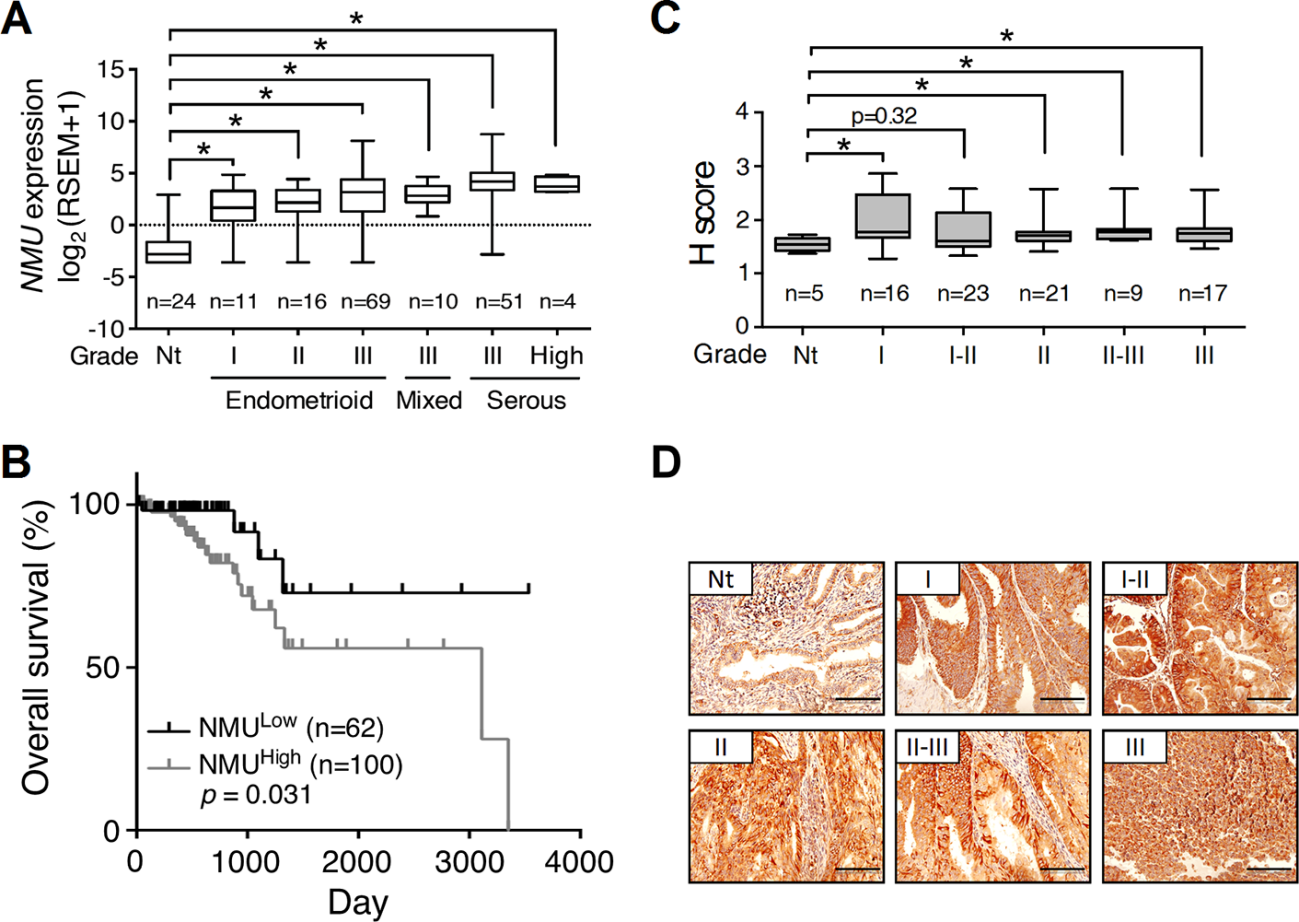
The expression profile of NMU and corresponding survival rate in human endometrial cancer (**A**) The values of *NMU* extracted from the TCGA RNA sequencing dataset were further log_2_ transformed by UCSC Cancer Genomics Browser and compared among normal tissues (Nt) and different grades and types of endometrial cancer. RSEM, RNA-Seq by Expectation Maximization. (**B**) Patient data were divided into NMU^low^ and NMU^high^ groups according to the mean of log_2_ transformed *NMU* values. The overall survival rates were then compared between these two groups. (**C**) Immunohistochemical analysis of NMU in the endometrial cancer tissue microarray. The signal intensities of NMU in the different grades of endometrial cancer were analyzed by the calculation of H score. For *A* and *C*, the boxes represent the interquartile range; the horizontal lines in the boxes represent the median; the whiskers represent the minimum and maximum. *p < 0.05. (**D**) Represented images from the sections of normal endometrium and each grade of endometrial cancer were presented. The morphology was revealed by counter-staining with hematoxylin. Scale bars, 100 μm.

The luminal epithelium of the uterine cavity is the major origin that gives rise to endometrial cancer. Using clinical samples from patients with endometrial cancer, we also confirmed the transcript level of *NMUR2* is higher than that of *NMUR1* especially in the high grade tumors (Supplementary Figure S2), consistent with the receptor profiles shown in the normal mouse uterus. We thus further explored the changes in NMU and NMUR2 profiles during human endometrial tumorigenesis. In the collected clinical samples, we preliminarily found that the transcript amounts of *NMU* were significantly augmented in the grades II and III, but not the grade I, tumor specimens as compared to those in their adjacent normal tissues (Supplementary Figure S3). More supports were given by analyzing the RNA sequencing data of uterine corpus endometrial carcinoma from The Cancer Genome Atlas (TCGA; http://tcga-data.nci.nih.gov/tcga/) *in silico* [7]. The gene profiles and patient characteristics were summarized in Supplementary Figure S4. In the TCGA cohort of 199 cases, the transcript level of *NMU* was significantly elevated in all types and grades of the primary tumors (Figure 1A and Supplementary Figure S4). Surprisingly, patients with the high *NMU* level have severe outcomes of low overall survival (Figure 1B) and low recurrence-free survival (Supplementary Figure S5). Our immunohistochemical staining results from a human endometrial tissue microarray further confirmed that the NMU protein signal was significantly elevated in the diverse grades of cancer as compared with normal tissues (Figure 1C and 1D). Regarding NMUR2, although our preliminary gene quantification data suggests that its transcript level is elevated in the high graded tumors (Supplementary Figure S3), neither the *NMUR2* expression data analyzed from the TCGA endometrial carcinoma cohort nor the NMUR2 protein staining summarized from an endometrial tissue microarray showed a significant difference between normal and cancer tissues (Supplementary Figure S6). Likewise, the high *NMUR2* level was not statistically correlated with overall survival (*p* = 0.182) and recurrence-free survival (*p* = 0.442) in patients. Taken together, our findings suggest that the *NMU* level may serve as a novel diagnosis marker when predicting the progression and disease outcome among endometrial cancer patients.

### NMU signaling controls the cell motility of the tested grade II endometrial cancer cells

**Figure 2:**
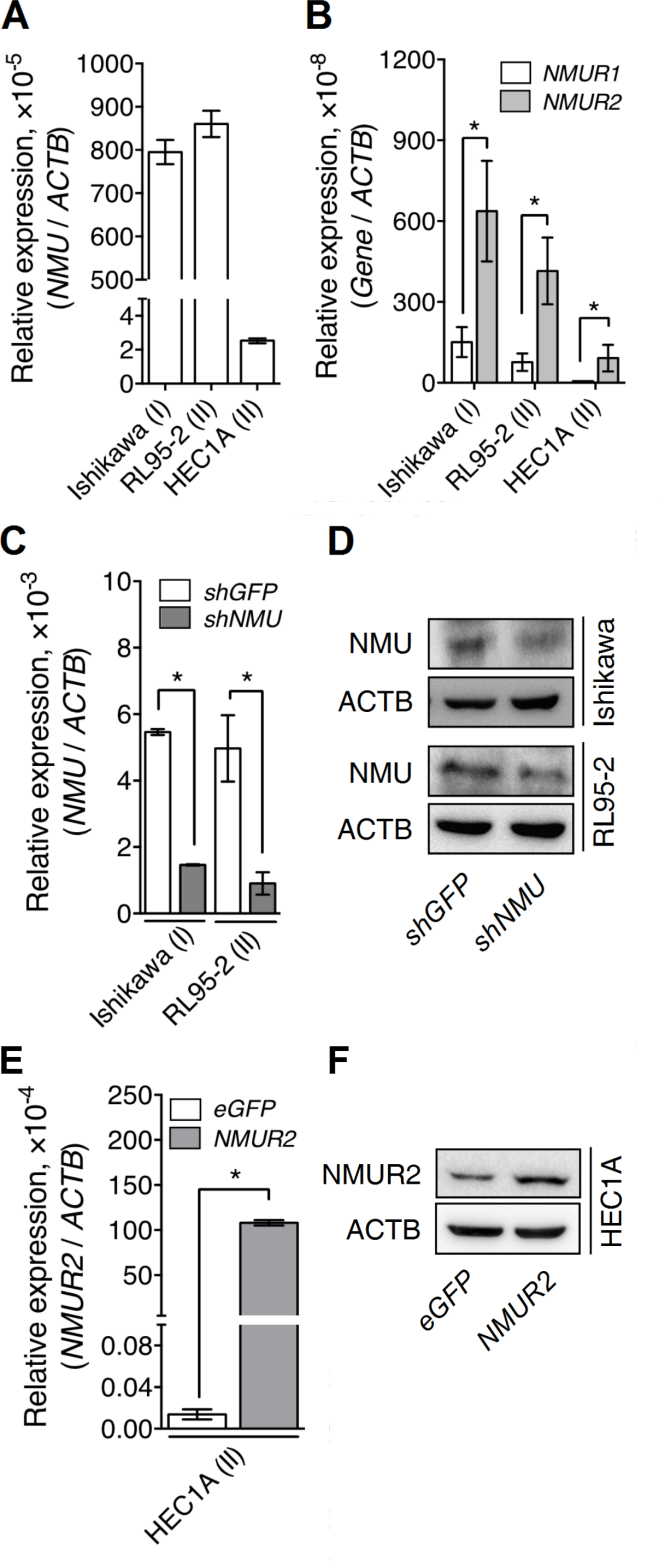
The endogenous and manipulated transcriptional profiles of *NMU* and NMURs in the various endometrial cancer cell lines The endogenous transcripts of (**A**) *NMU*, (**B**) *NMUR1* and *NMUR2* were determined in Ishikawa (from the grade I cancer), RL95-2 (from the grade II cancer) and HEC1A (from the grade II cancer). *NMU* knockdown efficiencies were evaluated by determining the changes in (**C**) the transcript and (**D**) the protein levels of NMU in Ishikawa and RL95-2. *NMUR2* overexpression efficiency in HEC1A was evaluated by determining the changes in (**E**) the transcript and (**F**) the protein levels of NMUR2. The transcript of each gene was quantified by real-time PCR and then normalized against *ACTB*. Data are shown as the mean ± SD. *p < 0.05.

To characterize the roles of NMU signaling during the progression of endometrial cancer, several cancer cell lines derived from patients with different grades of endometrial cancer, including Ishikawa (from grade I), RL95-2 (from grade II) and HEC1A (from grade II), were examined. We found that Ishikawa and RL95-2 exhibited much higher transcript levels of *NMU* and NMU receptors than HEC1A (Figure 2A and 2B). Furthermore, the level of *NMUR2* was much higher than that of *NMUR1* in all three endometrial cancer lines (Figure 2B), suggesting that NMUR2 is also the main receptor type involved in conducting NMU signaling in these cells. Based on the above, knockdown of *NMU* was applied to Ishikawa and RL95-2 whereas overexpression of *NMUR2* was applied to HEC1A to allow subsequent functional studies. The changes in the transcript and protein levels of these modified cells were validated by real-time PCR quantification and Western blotting (Figure 2C–2F).

**Figure 3:**
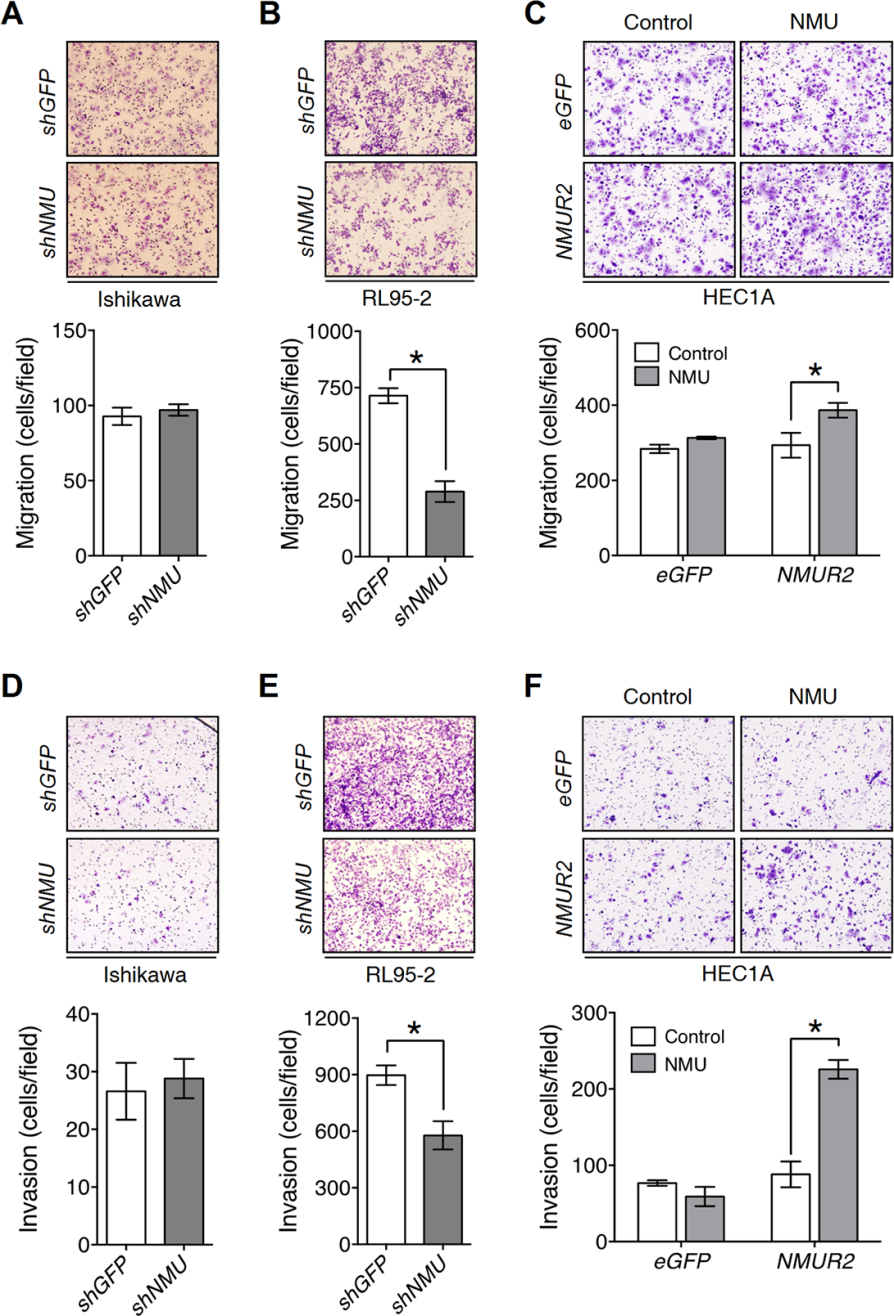
NMU signaling positively regulates cell motility in the grade II endometrial cancer cell lines Transwell migration assays were performed to evaluate NMU signaling-mediated migration ability in (**A**) Ishikawa, (**B**) RL95-2 and (**C**) HEC1A cells. Transwell chambers coated with Matrigel were used to assay NMU signaling-mediated invasiveness in (**D**) Ishikawa, (**E**) RL95-2 and (**F**) HEC1A cells. 100 nM NMU was used for the exogenouse NMU treatment of HEC1A cells. For all assays, a represented image was presented in the upper panel. To obtain the average cell number (lower panel), at least five fields of transmigrated cells were randomly photographed and counted for each individual experiment. The results are shown as the mean ± SD. *p < 0.05.

To investigate whether NMU signaling promotes the motility of endometrial cancer cells, Transwell migration and invasion assays were used. In contrast to negligible effects observed in Ishikawa, we found that knockdown of *NMU* significantly decreased the cell migration and invasion of RL95-2 (Figure 3A, 3B, 3D and 3E), but showed no effect on increasing cleaved caspase 3, a marker for apoptotic cells (Supplementary Figure S7A). In addition, overexpression of *NMUR2* in combination with NMU treatment promoted the cell motility, as well as the expression of matrix metalloproteinase 2, 3 and 9, of HEC1A (Figure 3C, 3F and Supplementary Figure S7B). Taken together, NMU signaling seems to have a profound effect on promoting the motility of the tested graded II endometrial cancer cells.

### The inhibition of proliferation in *NMU*-knockdown cells is accompanied by a morphological change

**Figure 4:**
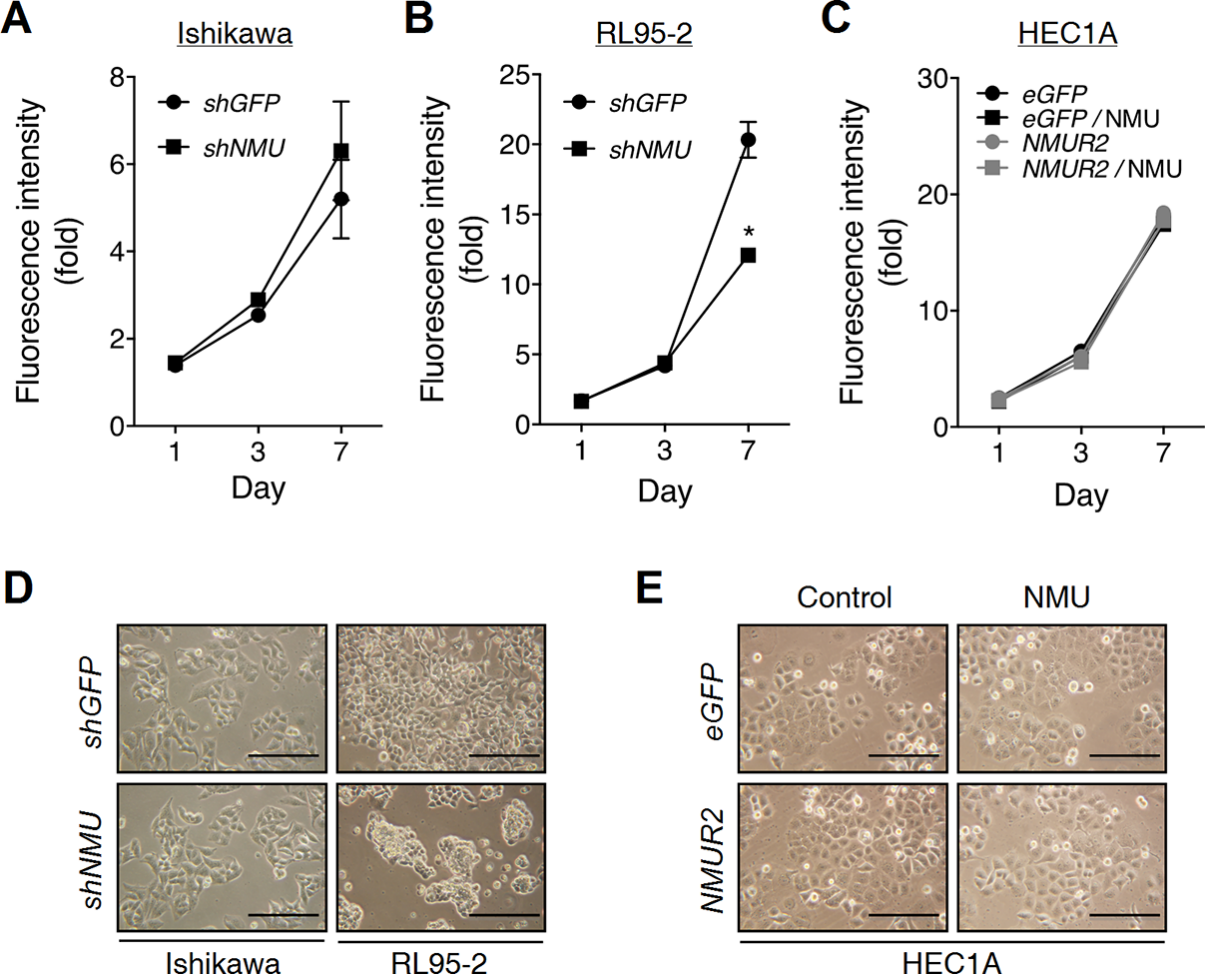
Effects of *NMU* knockdown on the cell growth and morphology of endometrial cancer cells (**A**) Ishikawa and (**B**) RL95-2 cells with or without *NMU* knockdown were cultured in the growth medium. (**C**) HEC1A cells with eGFP- or *NMUR2*-overexpression were cultured in the growth medium supplemented without or with 100 nM NMU. The growth rates were compared using the AlamarBlue assay. The fluorescence value of the cells on day 0 (D0) served as the one-fold control. Data are shown as the mean ± SD. *p < 0.05. The morphologies of (**D**) Ishikawa, RL95-2 and (**E**) HEC1A cells were observed on Day 5 of culture. Scale bar, 100 μm.

The effect of NMU signaling on endometrial cancer cell proliferation has not yet been explored before. In contrast to a negligible effect shown in *NMU*-knockdown Ishikawa or *NMUR2*-overexpressing HEC1A cells, knockdown of *NMU* in RL95-2 was found to significantly decrease cell proliferation in a long-term culture (Figure 4A–4C). By detecting the levels of P21 and cleaved caspase 3, the growth inhibition may be attributed to cell cycle arrest but not cell death (Supplementary Figure S8A).

Surprisingly, the decrease in proliferation was also accompanied by a change in cell morphology in *NMU*-knockdown RL95-2 cells by showing crowded cell clusters (Figure 4D and 4E). However, it seems that the cell-cell aggregation ability was not affected (Supplementary Figure S8B). In contrast, this crowded cell phenomenon has been also frequently reported in cells with a functional deficiency in adhesion molecules [23, 24], suggesting that the observed morphological change may be attributed to a decrease in cell-matrix adhesion, but not an enhancement in the cell-cell interaction.

### Depletion of NMU signaling decreases adhesion signaling and anchorage-independent growth in endometrial cancer cells

**Figure 5:**
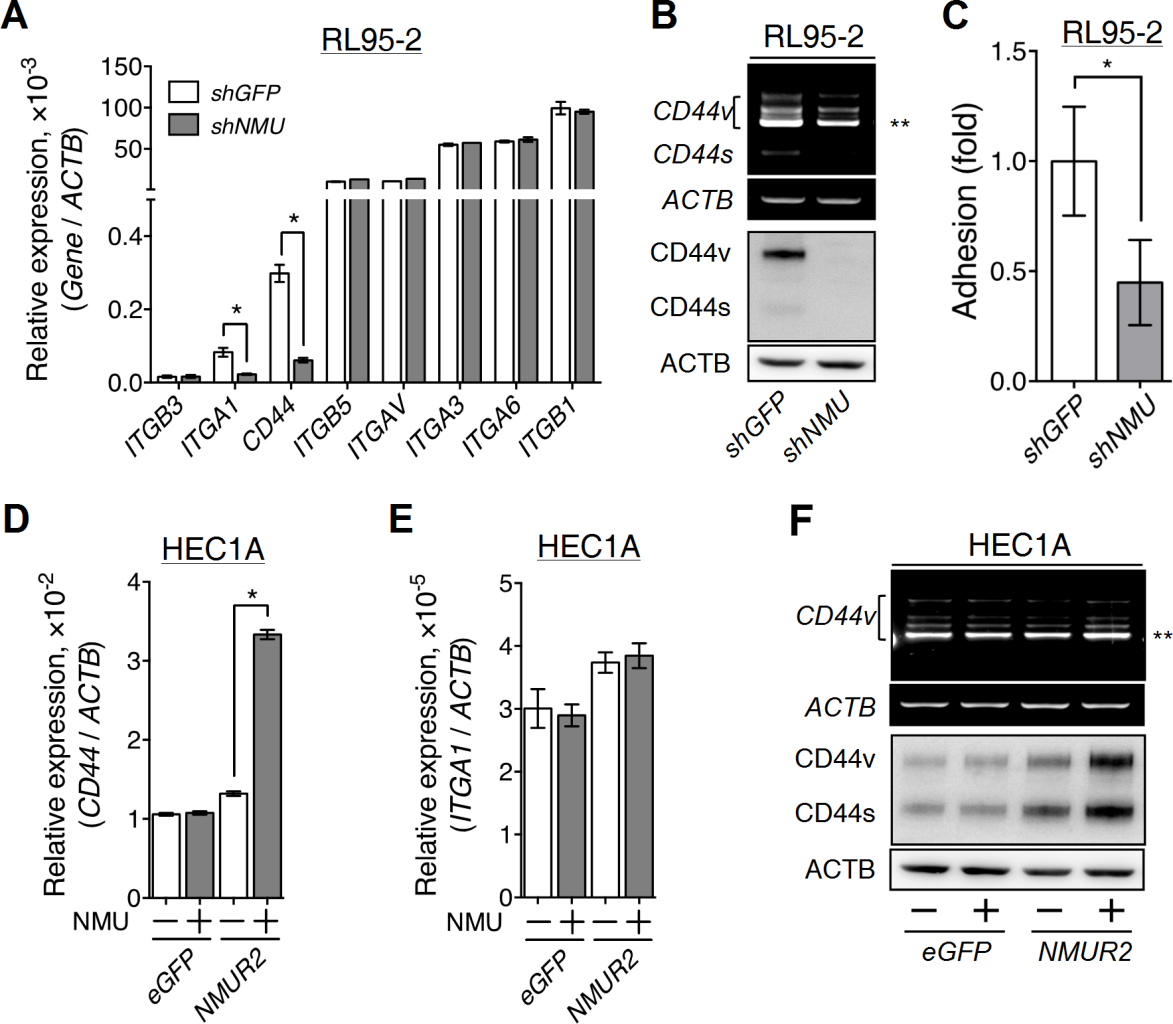
Effects of NMU signaling on the expression of adhesion molecules in the grade II endometrial cancer cell lines (**A**) The transcript levels of selected adhesion molecules were quantified in RL95-2 cells without or with *NMU* knockdown. (**B**) A primer pair across non-variable exons 5 and 7 of *CD44* (forward, CACCCCATCCCAGACGAA; reverse, CCACCTTCTTGACTCCCATGT) was used to unveil the transcript profiles of *CD44* splicing variants. The transcript profiles (upper panel) and protein profiles (lower panel) of standard CD44 (CD44s) and its splicing variants (CD44v) were revealed in RL95-2 cells without or with *NMU* knockdown. (**C**) RL95-2 cells without or with *NMU* knockdown were seeded on the hyaluronan-coated plate to compare their matrix adhesion ability. In addition, the transcript levels of (**D**) *CD44* and (**E**) *ITGA1*, and (**F**) the splicing profiles (upper panel) and protein profiles (lower panel) of CD44 were also compared between eGFP-overexpressing and *NMUR2*-overexpressing HEC1A cells without or with 100 nM NMU treatment. The transcript and protein levels of *ACTB* served as controls. Quantification data are shown as the mean ± SD. *p < 0.05. **CD44v8-10, the major splicing variant of *CD44* gene in RL95-2 and HEC1A cells.

To verify our hypothesis, we further investigated whether NMU signaling affects the expression of diverse adhesion molecules in endometrial cancer cells. After searching the gene expression profiles in the Cancer Cell Line Encyclopedia (CCLE) [25], several adhesion molecules abundant in RL95-2 were evaluated. Among them, the transcript levels of *ITGA1* and *CD44* were found to be decreased in *NMU*-knockdown RL95-2, with *CD44* showing the most dramatic difference (Figure 5A). CD44 exists as multiple isoforms that are generated through alternative splicing [26]. Using PCR in combination with sequencing, we verified that CD44v8-10 is the major transcript form of *CD44* (Figure 5B). Knockdown of *NMU* significantly decreased the transcript levels of all *CD44* variants, including CD44v8-10, as well as their protein amounts in RL95-2 cells (Figure 5B). Cell adhesion assay also confirmed that *NMU*-knockdown RL95-2 exhibited lower adhesion ability on a hyaluronan-coated dish (Figure 5C). Of interest, like *NMU*-knockdown cells, RL95-2 cells with *CD44* knockdown also showed phenotypes of crowded cell morphology and growth retardation (Supplementary Figure S9), suggesting that CD44 contributes a crucial role in mediating NMU signaling.

Furthermore, the reverse experiments using *NMUR2*-overexpressing HEC1A were done and the results indicated that only the transcripts and the protein amounts of various *CD44*, including the major form CD44v8-10, but not the *ITGA1* transcript, were increased by NMU treatment (Figure 5D–5F). Taken together, these data suggest that *CD44* may be a common gene that is positively regulated by NMU signaling in diverse endometrial cancer cells.

**Figure 6:**
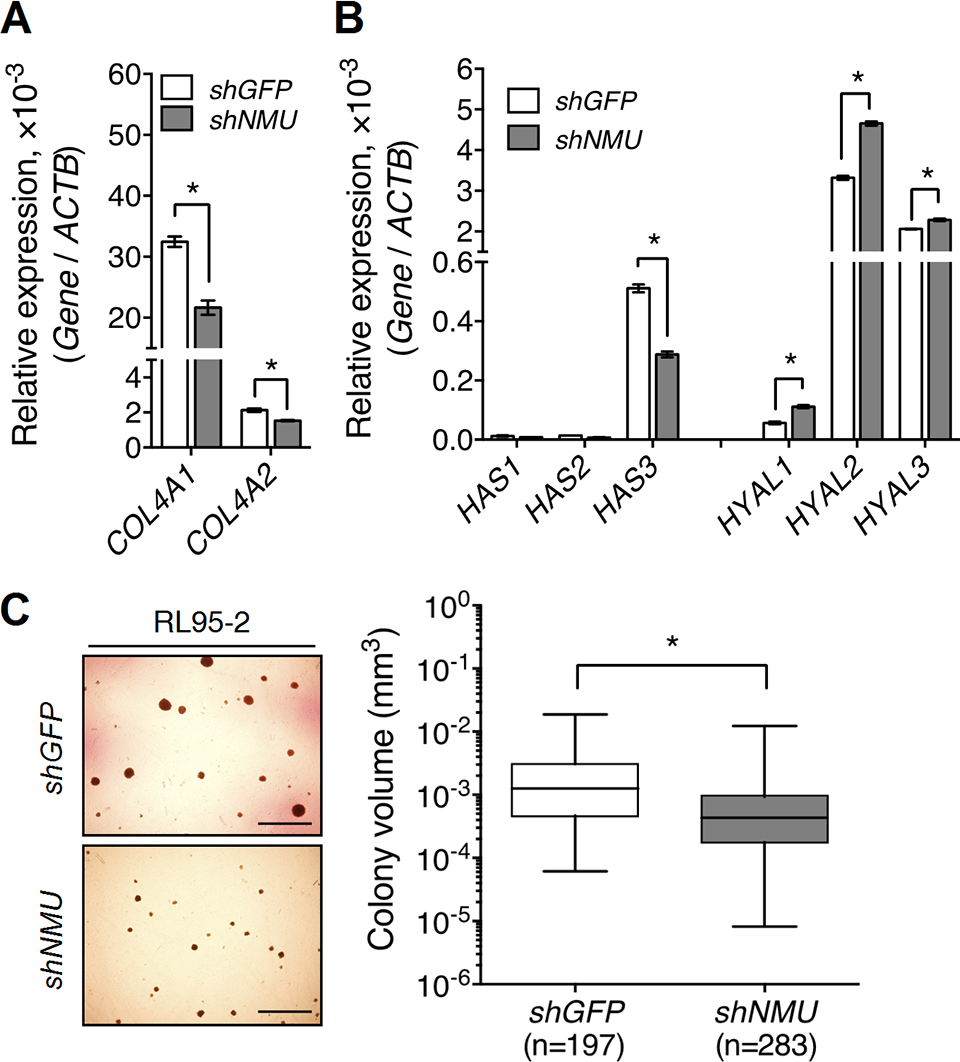
Knockdown of NMU decreases the expression of collagen IV and hyaluronan while at the same time reducing the anchorage-independent growth of RL95-2 cells The transcript levels of (**A**) type IV collagen genes, *COL4A1* and *COL4A2*, (**B**) hyaluronan production genes, *HAS1-3*, and hyaluronan degradation genes, *HYAL1-3*, were compared between control and *NMU*-knockdown RL95-2 cells. The transcript of each gene was quantified by real-time PCR and then normalized against *ACTB*. Data are shown as the mean ± SD. *p < 0.05. (**C**) Control and *NMU*-knockdown RL95-2 cells were cultured under anchorage-independent conditions for one month. The volume of each colony formed was calculated using the equation 4/3 × π × r^3^. The horizontal lines in each group represent the median. Scale bar, 200 μm *p < 0.05. Represented images were shown in the left panel.

In addition to promotion of the expression of integrins and *CD44*, we found that NMU signaling also increased the expression of their corresponding ligands. *NMU* knockdown decreased the transcript levels of *COL4A1* and *COL4A2*, which encode collagen IV, in RL95-2 (Figure 6A). *NMU* knockdown in RL95-2 also decreased the transcript level of *HAS3*, the enzyme that promotes hyaluronan production; there were also increases in the transcript levels of *HYAL1*, *HYAL2* and *HYAL3*, the enzymes involved in hyaluronan degradation (Figure 6B). Thus, the impairment of anchorage signaling may explain the growth delay and morphological change shown in *NMU*-depleted RL95-2 cells. Not only this, endogenous hyaluronan produced by cancer cells themselves has been proposed to participate in anchorage-independent growth [27]. Indeed, *NMU* depletion dampened the anchorage-independent growth ability of RL95-2, as shown by a significant 3-fold reduction in the average size of the colonies formed (Figure 6C).

### NMU pathway controls the motility and proliferation of endometrial cancer cells through adhesion signaling

**Figure 7:**
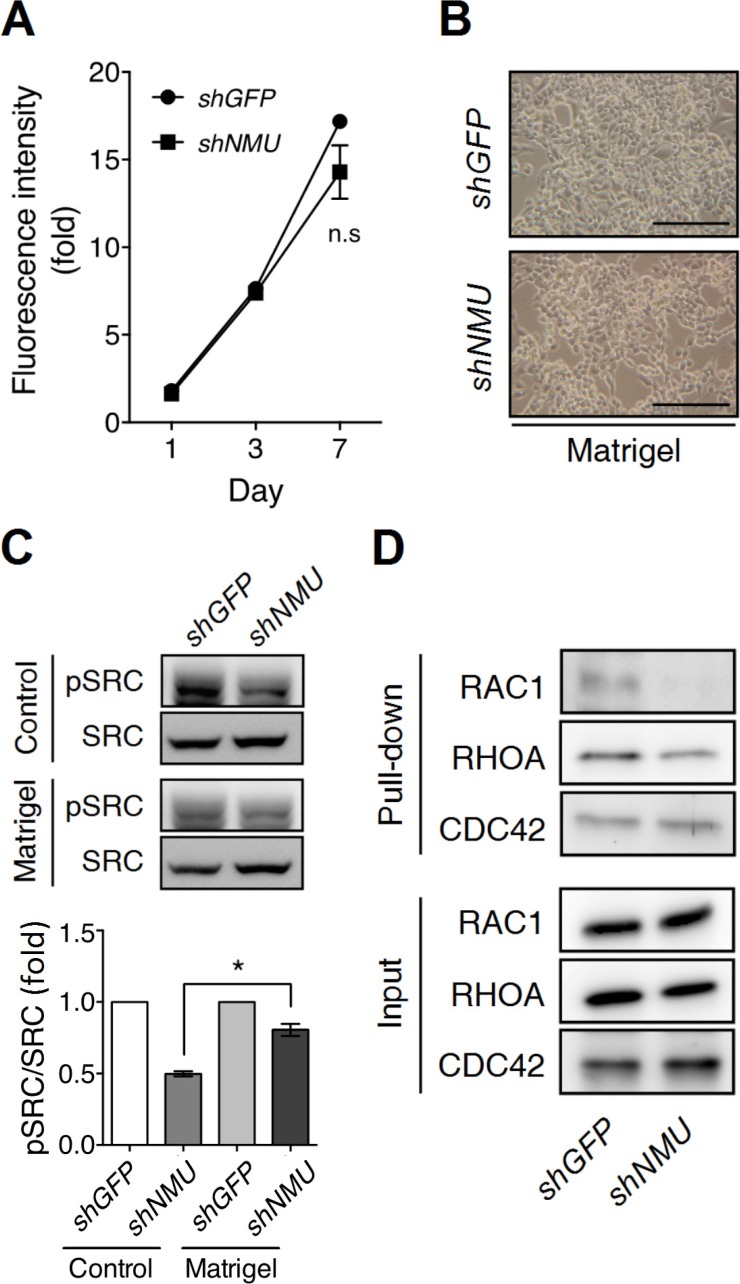
Matrigel culture remedies the effects of *NMU* knockdown in RL95-2 cells RL95-2 cells without or with *NMU* knockdown were seeded onto the Matrigel-coated dishes for culture. (**A**) The growth rates were compared using the AlamarBlue assay. The fluorescence value on day 0 (D0) served as the one-fold control. The results are shown as the mean ± SD. n.s, no significance. (**B**) Represented images of cell morphology were shown on Day 5 of culture. Scale bar, 100 μm. (**C**) In the upper panel, the levels of phospho-SRC protein in GFP-knockdown control or *NMU*-knockdown cells seeded on the standard or Matrigel-coated dishes were compared by Western blotting on Day 5 of culture. Total SRC protein served as the internal control. In the lower panel, the band intensities were further quantified by densitometer in three individual experiments. Fold changes in the phospho-SRC/total SRC level between cells seeded on the standard and Matrigel-coated dishes were normalized and compared. Data are shown as the mean ± SEM. *p < 0.05. (**D**) The active forms of RAC1, RHOA and CDC42 under the above conditions were pulled down by their affinity beads for Western blotting. The inputs of RAC1, RHOA and CDC42 served as loading controls.

Next we investigated how NMU pathway controls adhesion signaling. Surprisingly, when plated on the Matrigel-coated dishes, *NMU*-knockdown RL95-2 showed no difference in either cell morphology or proliferation rate when compared with control cells under a long-term culture (Figure 7A and 7B). These findings suggest that Matrigel-derived adhesion signaling is able to compensate for the signaling loss caused by *NMU* depletion.

SRC family kinases are known to be central components of cellular adhesion responses [28]. In fact, SRC family kinases have long been proposed to participate in hyaluronan/CD44-driven cellular events [29, 30]. Indeed, we observed a significant decrease in the amount of activated c-SRC present in *NMU*-depleted RL95-2 (Figure 7C). Of interest, administration of a SRC family kinase inhibitor to the control RL95-2 cells can totally mimic the *NMU*-knockdown phenotypes as shown by the crowded cell-clustering morphology and the growth inhibition (Supplementary Figure S10). In contrast to the above, the c-SRC phosphorylation level in *NMU*-knockdown RL95-2 can be restored when the cells were cultured on the Matrigel-coated dishes (Figure 7C). Furthermore, the activity levels of Rho GTPases, which are downstreams of SRC kinases, were also investigated. It was found that knockdown of *NMU* decreased the levels of activated RAC1 and RHOA, but not that of CDC42, in RL95-2 cells (Figure 7D). Thus, the findings suggest that activation of NMU signaling can increase adhesion signaling and thence cell spreading and growth through SRC and Rho GTPase-dependent pathways.

### NMU signaling facilitates growth factor-driven EMT in endometrial cancer cells

**Figure 8:**
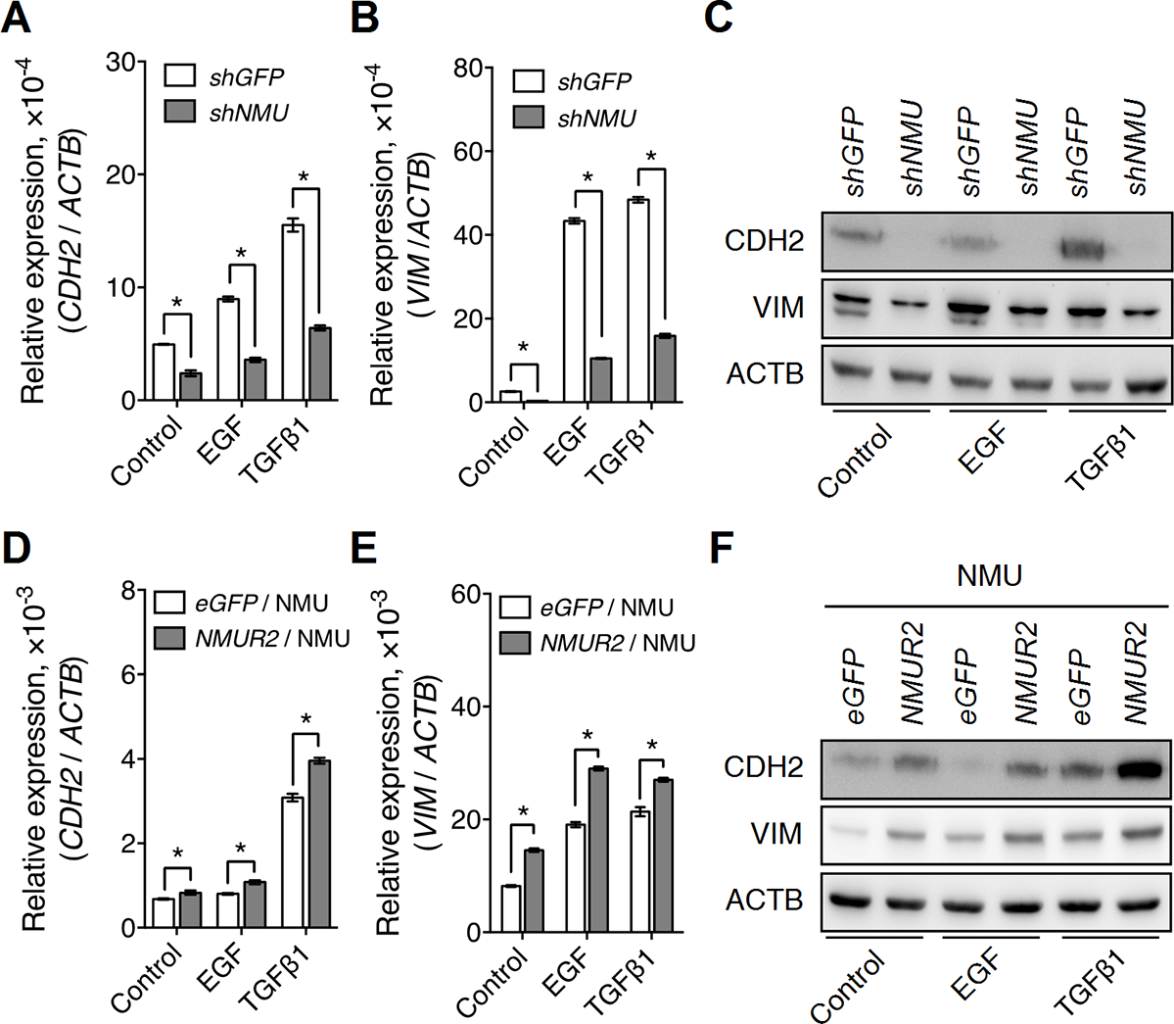
*NMU* signaling positively regulates growth factor-driven mesenchymal marker expressions in the grade II endometrial cancer cell lines The transcript levels of (**A**) *CDH2* (N-cadherin) and (**B**) *VIM* (vimentin), and (**C**) the protein levels of CDH2 and VIM were compared between control and *NMU*-knockdown RL95-2 cells in the absence or presence of EGF (10 ng/ml) or TGFβ1 (3 ng/ml) treatment. In addition, the transcript levels of (**D**) *CDH2* and (**E**) *VIM*, and (**F**) the protein levels of CDH2 and VIM were compared between eGFP-overexpressing and *NMUR2*-overexpressing HEC1A cells treated with 100 nM NMU in the absence or presence of EGF or TGFβ1. The transcript levels from real-time PCR were normalized against *ACTB* and are shown as the mean ± SD. *p < 0.05. The corresponding proteins were detected by Western blotting using ACTB as the internal control.

Intriguingly, CD44 has also been reported to function as a co-receptor for several growth factor receptors, such as EGFR and TGFβ receptor [16]; their coupling is believed to be able to enhance the growth factor-driven tumor proliferation and/or metastasis. In our finding, knockdown of *NMU* in RL95-2 was found indeed to attenuate the effect of EGF or TGFβ on the induction of mRNA and protein expressions of N-cadherin and vimentin (Figure 8A–8C). Of interest, similar effects were also observed in RL95-2 cells with *CD44* knockdown (Supplementary Figure S11). In addition, these responses were further confirmed in the reverse experiment when *NMUR2*-overexpressing HEC1A cells were treated with NMU (Figure 8D–8F). Taken together, our data suggest that activation of NMU signaling can positively contribute to the enhancement of growth factor-driven mesenchymal transition of endometrial cancer cells and that this potentially happens by sustaining the expression levels of CD44.

### Depletion of NMU signaling dampens tumorigenesis in endometrial cancer cells *in vivo*

**Figure 9:**
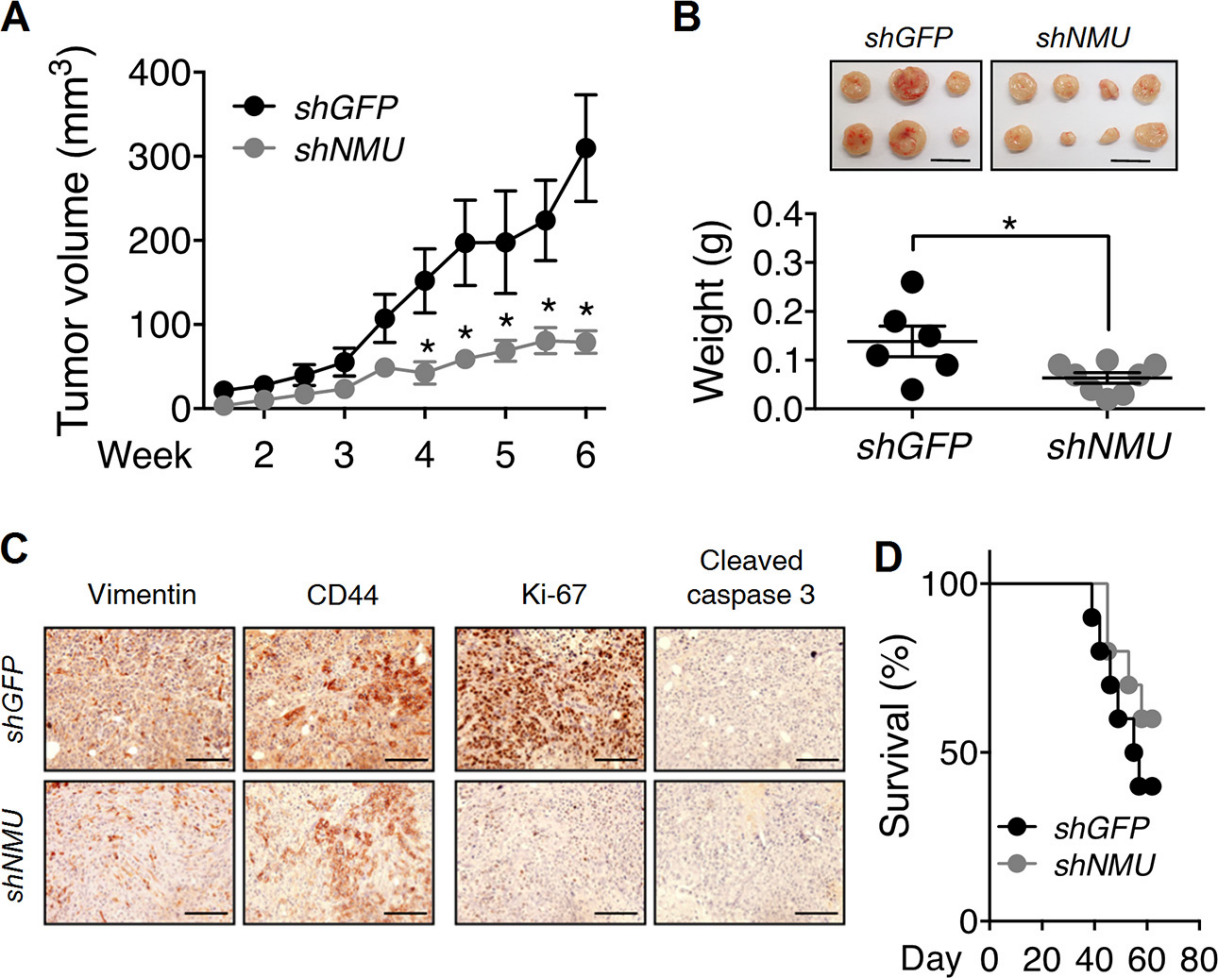
Effects of NMU knockdown on tumor growth and animal survival rate *in vivo* (**A**) Control RL95-2 and *NMU*-knockdown RL95-2 cells were subcutaneously injected into nude mice and the sizes of the tumors formed at indicated intervals were measured. (**B**) The weights of harvested tumors at the end of experiments were measured. The results are shown as the mean ± SEM. *p < 0.05. Scale bar, 1 cm. (**C**) Immunohistochemical analyses against vimentin, CD44, Ki-67 and cleaved caspase 3 were performed. Cell morphology was revealed by counter-staining with hematoxylin. Scale bars, 100 μm. (**D**) Control RL95-2 and *NMU*-knockdown RL95-2 were intraperitoneally injected into nude mice and the survival rates of the mice at indicated intervals were monitored (*n* = 10).

Based on the above, NMU signaling is able to control cell motility and proliferation *in vitro*, potentially by increasing the adhesion capability and growth factor sensitivity of the cells. Therefore, the effects of NMU signaling on endometrial tumorigenesis *in vivo* were further evaluated. Subcutaneous injection using the nude mouse model indicated that mice bearing with *NMU*-knockdown RL95-2 cells started to show a significant reduction in the average tumor volume after 4 weeks as compared to mice injected with control RL95-2 cells (Figure 9A). At the end of the experiment (6 weeks), it was found that knockdown of *NMU* significantly retarded tumor weight by 50% on average (Figure 9B). As compared with the control tumors, immunohistochemical staining confirmed that the tumors derived from *NMU*-knockdown RL95-2 cells exhibited an apparent reduction in the mesenchymal marker vimentin as well as a moderate reduction in CD44 (Figure 9C), consistent with the cell line results *in vitro*. These differences were also accompanied by a significant decrease in the staining intensity of the proliferation marker Ki-67. In contrast, the staining of the cell death marker cleaved caspase 3 remained low and comparable with control tumors (Figure 9C). Therefore, the weight reduction in *NMU*-depleted tumors may be due to a decrease of cell proliferation ability and was not due to promotion of cell death.

In addition, intraperitoneal injection into nude mice was used to mimic the metastatic dissemination of endometrial cancer in the abdominal cavity. Although not significant (*p* = 0.3), the results indicated that nude mice injected with *NMU*-knockdown RL95-2 cells showed a potential increase in the survival rate as compared with those injected with control cells (Figure 9D).

## DISCUSSION

To our knowledge, this is the first study to characterize the uterine profile, the tumorigenic effects and also the underlying mechanisms of NMU signaling during endometrial cancer progression. We identified NMUR2 as the major type of NMU receptors present in the uterus, where it co-expresses with NMU mainly in the uterine endometrium. Not only this, we also demonstrated that NMU signaling is directly involved in promoting endometrial cancer malignancy in the tested cell lines isolated from the grade II cancer. However, we also noticed that knockdown of *NMU* in Ishikawa cell line, from the grade I cancer, did not change various tumor progression-related events, suggesting that the involvement of NMU signaling in endometrial tumorigenesis might be heterogeneous and depend on the cancer stages. Indeed, we observed that the transcript level of NMU is correlated with the malignancy and the low survival rate of endometrial cancer. Therefore, it is concluded that NMU might be useful as a diagnosis marker when predicting the progression and disease outcome among endometrial cancer patients.

Our findings also indicated that NMU signaling is able to sustain the expressions of adhesion molecules such as CD44 and hyaluronan. With CD44 playing as an auxiliary receptor in promoting growth factor receptor activities [15, 16], this explains how NMU signaling also tightly controls the intensity of EGFR-driven and TGFβ receptor-driven EMT in endometrial cancer cells. The mechanism by which NMU signaling acts as a key allocator to coordinate the growth factor receptor cascades is absolutely novel. Of interest, NMU signaling has been proposed to be involved in the progression of several other types of cancers. For example, NMU signaling can promote cell growth in acute myeloid leukemia, bladder cancer, non-small-cell lung cancer and breast cancer [31–34]. NMU signaling is also capable of increasing cell motility in pancreatic caner and renal cancer *in vitro*, and of promoting metastasis in bladder cancer and breast cancer *in vivo* [32, 34–36]. However, the underlying mechanisms through which these cancer events are controlled have not yet been characterized. Therefore, it will be interested to verify whether our findings can be applied to other types of cancers that have been shown to have NMU signaling involvement.

In the present study, we demonstrated that NMU signaling not only can promote the expression levels of both receptors and ligands contributing to adhesion signaling, but may also increase the activities of SRC family kinases, such as c-SRC, in endometrial cancer cells (Figures 5–7). SRC family kinases have long been known to act as the downstream effectors within the adhesion signaling cascades and exert a profound effect on many tumor progression-related events [37–39]. In consequence of the stimuli from adhesion signaling, activated SRC family kinases will lead to increases in cell adhesion, spreading, migration and proliferation; these events occur via the activity controls of both guanine nucleotide-exchange factors and GTPase-activating proteins that act on Rho-GTPases [28]. This also helps to explain why in our study depletion of *NMU* in RL95-2 cells leads to a reduction in activated RAC1 and RHOA (Figure 7D).

Of interest, not only correlated with their CD44 co-receptor level, the intensity of ERBB and TGFβ receptor signaling cascades can also be directly controlled by SRC family kinases. Taking EGFR as an example in ERBB, phosphorylation of EGFR at Tyr845 and Tyr1101 directly by c-SRC helps EGFR to retain strong activity [40]. Abolition of SRC kinases-mediated EGFR phosphorylation has been shown to inhibit EGF-induced proliferation and/or transformation in many types of cancer cells, such as breast cancer cells and cervical cancer cells [41, 42]. As to TGFβ receptor signaling, it has been shown that c-SRC can phosphorylate TGFβ type II receptor directly at Tyr284 and this controls the TGFβ-driven activation of p38 MAPK and induction of EMT, which in turn lead to a promotion of proliferation and invasion in breast cancer cells [43]. In our studies in *NMU*-depleted endometrial cancer cells, we observed that not only was there a decrease in the levels of adhesion signaling-related molecules, but there was also an impairment of downstream c-SRC activity (Figures 5–7). Therefore, we can not exclude the possibility that the impairment of SRC activity can contribute directly, or has a synergistic effect with the decrease of CD44 co-receptor level, to dampen the EGF-induced or TGFβ1-induced mesenchymal marker expression in the *NMU*-knockdown endometrial cancer cells (Figure 8).

Taken as a whole, from the view of the cancer microenvironment, an up-regulation of NMU signaling in progressive endometrial tumor cells would also help these malignant cells become more sensitive to niche growth factors such as EGF and TGFβ via the adhesion signaling-SRC cascades. Therefore, combined therapy that includes targeting of NMU signaling may enhance the efficacy of current drugs that are designed to inhibit EGFR and/or TGFβ receptor pathways; such an approach could be beneficial when treating patients with aggressive endometrial cancer.

## MATERIALS AND METHODS

### Ethical statements and clinical materials

The specimens for transcript quantification were obtained from the patients with endometrial cancer who underwent surgery at Chang Gung Memorial Hospital Linkou Medical Center. Written informed consent was signed and obtained from all participants. The study protocol was approved by the research ethics committee of Chang Gung Memorial Hospital Linkou Medical Center, Chang Gung University (approval number 104-5509B). The tumors were graded according to the FIGO classification [44]. Five grade I, two grade II and four grade III of endometrial cancer specimens and their paired adjacent normal tissues were included.

The endometrial cancer tissue microarrays were purchased from Pantomics and US Biomax (catalog number EMC1021). More than 80% patients are above 45 years old and the mean age of patients is 53 years old. Several cores with tissue missing or inappropriate staining were excluded from evaluation. The RNA sequencing data of uterine corpus endometrioid carcinoma from TCGA were processed and analyzed by the Cancer Genomics Browser (ID: TCGA_UCEC_exp_HiSeqV2_PANCAN) (https://genome-cancer.ucsc.edu) [45].

### Animal ethics and treatments

ICR mice and BALB/c nude mice were purchased from BioLASCO (Taipei, Taiwan). All the *in vivo* experiments were approved by the Institutional Animal Care and Use Committee of the National Yang-Ming University (approval number: 990504). The uteri in different stages of the estrous cycle were collected from ICR mice based on the protocols described previously [46, 47].

For the tumor growth assay *in vivo*, 5 ×10^6^ tumor cells were subcutaneously administrated into 7-week-old female BALB/c nude mice. The tumor volume was calculated by the formula (0.5 × length × width^2^) at indicated intervals. At the endpoint of experiments, the weight of isolated tumors was measured. For the survival assay, 2 × 10^7^ tumor cells were intraperitoneally injected into BALB/c nude mice. The survival rates of tumor-injected nude mice were evaluated.

### Immunohistochemistry

The processes of tissue preparation, section and staining have been described previously [22]. The signals were visualized using the NovaRed HRP substrate kit (VECTOR, Burlingame, CA). The sections were counterstained with hematoxylin.

To evaluate the expression levels of NMU and NMUR2 proteins in the endometrial cancer tissue microarray, the averaged staining intensity in each core of the tissue microarray was quantified and scored as 0, 1, 2 or 3. The H score of each core was calculated by multiplying the intensity score by the percentage of positive staining in each core.

### cDNA preparation and gene quantification

Total RNAs isolated from the mouse uteri, endometrial cancer specimens or endometrial cancer cell lines were further used for cDNA synthesis with oligo dT primer. Gene quantification was performed by Power SYBR^®^ Green PCR Master Mix (Life Technologies, Carlsbad, CA) and calculated by the 2^(−ΔCt)^ formula. The primer pairs used for gene quantification were shown in Supplementary Table S1.

### Cell lines and plasmids

Ishikawa cell, originally purchased from the European Collection of Cell Culture, was kindly provided by Dr. Sin-Tak Chu. RL95-2 and HEC1A cells were originally obtained from ATCC. For gene manipulation, the cDNA encoding eGFP or human NMUR2 was amplified and subcloned into the pLAS2w.Ppuro lentivector. The shGFP-expressing lentivector, sh*NMU*-expressing lentivector, sh*CD44*-expressing lentivector and other plasmids used for the lentivirus production and establishment of stable cell lines were purchased from National RNAi Core Facility Platform, Taiwan. Infected endometrial cancer cells were selected and maintained in the puromycin-containing medium.

### Migration and invasion assays

Transwell chambers (8 μm-pore size) without or with 250 μg/ml Matrigel coating were respectively used for the migration assay or the invasion assay. 3 × 10^5^ Ishikawa, 1 × 10^6^ RL95-2 or 2 × 10^5^ HEC1A cells were resuspended with serum-free medium and placed into the upper chambers. Growth medium was added to the lower chambers as chemoattractants. For HEC1A, 100 nM NMU peptide was added in the upper and lower chambers. After 24 hours, the migrated or invaded cells were fixed and stained by Giemsa Stain for subsequent counting.

### Proliferation and anchorage-independent growth assays

For the proliferation assay, 1 × 10^3^ Ishikawa, 1 × 10^3^ RL95-2 or 3 × 10^3^ HEC1A cells were seeded in 48-well plates pre-coated without or with 20 μg/ml Matrigel. At specific intervals, the cells were further incubated with 10% medium volume of AlamarBlue (AbD Serotec, Oxford, UK) for 3 hours at 37°C followed by detection of fluorescence intensities.

For the anchorage-independent growth, 5 × 10^3^ RL95-2 cells were resuspended with growth medium containing 1% methylcellulose and seeded in 3.5-cm dishes that were pre-coated with 30 mg/ml polyhydroxyethylmethacrylate. After 1 month, the colonies were photographed for size measurements.

### Aggregation and adhesion assay

StemPro Accutase Cell Dissociation Reagent (Life Technologies) was used to dissociate cells in the aggregation assay and adhesion assay. For the aggregation assay, control and *NMU*-knockdown RL95-2 cells were resuspended with ice-cold growth medium. 1 × 10^5^ cells were seeded in the PCR tube and then centrifugated at 1000 rpm for 5 min at 4°C followed by further incubation at 37°C for indicated intervals. Cell clusters were pipetted 10 times following paraformaldehyde fixation for 15 min at 4°C. The number of cell particle was counted by hemocytometer. The aggregation index at indicated intervals were calculated by the equation (N_0_-N_t_)/N_0_.

For the adhesion assay, control and *NMU*-knockdown RL95-2 cells were resuspended by serum-free medium containing 0.5 μg/ml Calcein AM (Life Technologies). 1 × 10^5^ cells were seeded on the 96-well plate precoated by 1% heat-denatured BSA, 100 μg/ml poly-L-lysine or 8 mg/ml hyaluronan hydrogel (HyStem Cell Culture Scaffold Kit, Sigma) and further incubated for 2 hours at 37°C. Wells were further filled to the brim by serum-free medium and sealed by adhesive plate seals. The plate was then inverted and the non-adhesive cells were dislodged by centrifugation at 150 g for 5 min at 4°C. The area of adhesive cells was quantified by fluorescence microscope. The cell adhesion index was calculated by the equation (Area_hyaluronan_ - Area_BSA_)/(Area_poly-L-lysine_).

### Rho GTPase activation assay

The RHOA/RAC1/CDC42 Activation Assay Combo Biochem Kit (Cytoskeleton, Denver, CO) was used according to the manufacturer instructions. Briefly, 3 × 10^6^ RL95-2 cells were seeded in 10-cm dish for 6 days. The cells were washed with ice-cold PBS and then collected with lysis buffer. 600 μg/ml total lysate was further incubated with specific GTPase affinity beads at 4°C for 1.5 hours. The activated GTPases were eluted for Western blotting.

### Statistical analysis

The data from gene quantification, cell proliferation, migration and invasion are shown as the mean ± SD. At least three individual repeated experiments were carried out, and these showed similar results. Statistical significance in individual group was determined by the Student's *t*-test. The data from *in vivo* mice experiments are shown as the mean ± SEM; statistical significance was analyzed by the Mann-Whitney test. Statistical significance was analyzed by the ratio paired *t*-test for comparing the gene expression between cancer and adjacent normal tissues. Statistical differences in the expression of NMU and NMU receptors in cancer tissues and in the H scores of the tissue microarray were analyzed by the Mann-Whitney test. Statistical significance was analyzed by Log-rank test for the survival curves.

## SUPPLEMENTARY MATERIALS TABLE AND FIGURES


