# Supplementary material for: NMU signaling promotes endometrial cancer cell progression by modulating adhesion signaling
Source: Oncotarget. 2016 Feb 3;7(9):10228–42. doi: 10.18632/oncotarget.7169 (PMC4891116; doi:10.18632/oncotarget.7169)
Supplement: Supplementary file 1 [file oncotarget-07-10228-s001.pdf]

# NMU signaling promotes endometrial cancer cell progression by modulating adhesion signaling

## Supplementary Materials

### Supplementary Table S1: Primer list for real-time quantitative PCR

| Gene | Species | Forward primer | Reverse primer |
|---|---|---|---|
| *Nmu* | Mouse | CAGAACAAGAACTACAATTGTGGA | AAGCTTCCTCAATGCAACAGAT |
| *Nmur1* | Mouse | GGAGGTCAAGGGCAGGAAA | GATGCCGAATACCACAACCA |
| *Nmur2* | Mouse | TGTTCTTGCCCTGTGGTGTA | CGGTGGCAAGTGCTTTTGT |
| *NMU* | Human | CTGCCGAGGTGCTCCAATA | CAATGGACAGAAAAGACGAACA |
| *NMUR1* | Human | GCCGGAGACAAGTGACCAAGA | TGACACGACGCTCCACATG |
| *NMUR2* | Human | CCTATTCTACCTCCTCCCCATGAC | CATTCCCTTCATCTGCCTCAA |
| *CDH1* | Human | ATGAGTGTCCCCCGGTATCTT | CAGTATCAGCCGCTTTCAGATTTT |
| *CDH2* | Human | CGCCAGGCCAAACAACTT | TCTCCTCCACCTTCTTCATCATATTT |
| *VIM* | Human | CGCCAGATGCGTGAAATG | CAGGCGGCAATAGTGTCTT |
| *TWIST1* | Human | ACGCTGCCCTCGGACAA | CCATCCTCCAGACCGAGAAG |
| *SNAI1* | Human | CCTGTCTGCGTGGGTTTTTG | GAGTCTGTCAGCCTTTGTCCTGTA |
| *SNAI2* | Human | GCCTCCAAAAAGCCAAACTACA | AGCAGCGGTAGTCCACACAGT |
| *CD44* | Human | GACAGGCTCACTCAAGCTCTTTAAC | CAGGTAGAGGCTGTTGTAACCATTT |
| *ITGA1* | Human | GTATGCTCTCAATCAGACAAGGTTT | CCCCGCATGGCTCATT |
| *ITGA3* | Human | CAGACCCAACAGCAAAGGAA | GCCGTTTGCCTGTCTTTGT |
| *ITGA6* | Human | TTTTTGGCGTGGCTGACTTA | TGCCTCCTCAGCTACACTATCTG |
| *ITGAV* | Human | ATGAGCTTGGTGGAGAAGAATGA | AAGGTGACATTGAGATGGGTAGTG |
| *ITGB1* | Human | ATTTTGTTTAATGTCTGGTGCTTTCTG | TTACCCCAAAATTGCAAACAAATAC |
| *ITGB3* | Human | TCTCACCCATGGATTACTCCTTACT | CTCCCCGCCCCCTATTT |
| *ITGB5* | Human | GACTTTTCTGCGTGATGGCTAT | ACGGACAGGAGAGGAAACTGA |
| *COL4A1* | Human | CTACGCCGTCCACCTTGAA | TTTCTAGGGTTCGTTGCTGTTA |
| *COL4A2* | Human | GCCGCTGTTCTGTGTGTGA | TGTGCATGAGGAAGGAATATCC |
| *HAS1* | Human | CCCCGAGCCACGAACTT | TGACCCCCTCGTTTTGCA |
| *HAS2* | Human | GAAACAGCCCCAGCCAAA | AAGACTCAGCAGAACCCAGGAA |
| *HAS3* | Human | TGGCCCTCCTACTGCTATATCT | TCCCCTTCCCTCCCTTACC |
| *HYAL1* | Human | ACCAGCCACCCCAAAGC | CATTTGAACTCCACAGCCATCT |
| *HYAL2* | Human | CGCAACTGGCAGGACAA | GAACTGCTGTGCTGCGAACT |
| *HYAL3* | Human | TGTCCCACTCTTGCTCTATTCAG | CGTTTTTTCTGGCCCCTTCT |
| *MMP2* | Human | CCGCAGTGACGGAAAGATGT | CACCTTGCGGTCATCATCGTA |
| *MMP3* | Human | CCAAATGCAAAGAAAGTGACACA | GTGCCTTCTACATATCTCTTTCAACAA |
| *MMP9* | Human | GGACGATGCCTGCAACGT | CAAATACAGCTGGTTCCCAATCT |

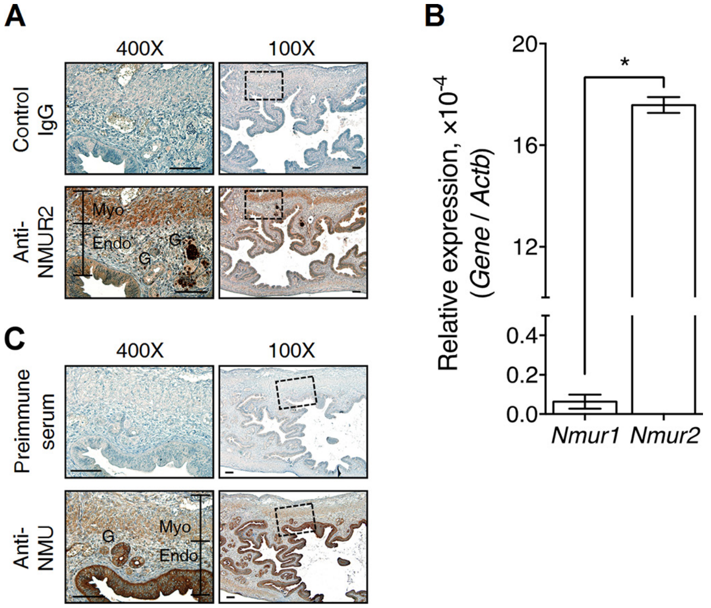


**Supplementary Figure S1: The expression profiles of NMU and NMU receptors in the mouse uterus.** Immunohistochemical staining against (**A**) NMUR2 (upper panel) or (**C**) NMU (lower panel) was performed in the estrus stage of the mouse uterus. Control IgG and preimmune serum from rabbits served as the staining controls for NMUR2 and NMU, respectively. The morphology was revealed by counter-staining with hematoxylin. G, gland. Endo, endometrium. Myo, myometrium. Scale bars, 100 µm. (**B**) The transcripts of *Nmur1* and *Nmur2* in the mouse uterus in the estrus stage were quantified by real-time PCR. Data were normalized against *Actb* and are shown as the mean ± SD. *$p < 0.05$.

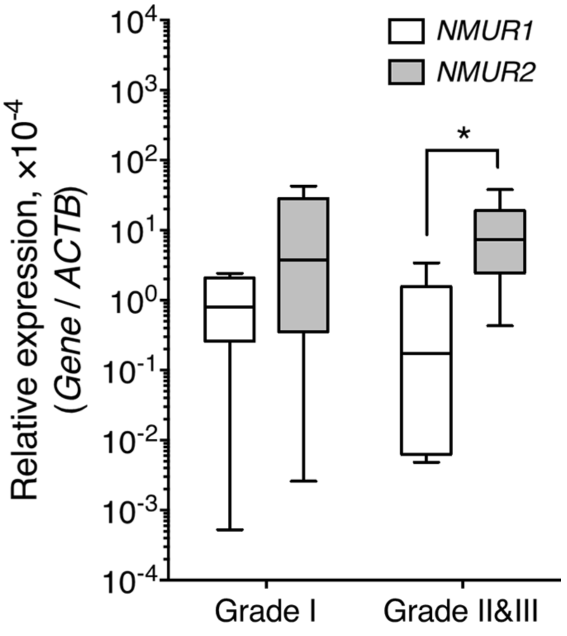


**Supplementary Figure S2: The transcript profiles of *NMUR1* and *NMUR2* in the cancer tissue parts of human endometrial cancer.** The transcript levels of *NMUR1* and *NMUR2* in the endometrial cancer tissue parts of the grade-I group ($n = 5$) and the grade-II & III groups ($n = 6$) were compared. Data were first quantified by real-time PCR and then normalized by the corresponding *ACTB* level for each specimen. The boxes represent the interquartile range; the horizontal lines in the boxes represent the median; the whiskers represent the minimum and maximum. *$p < 0.05$.

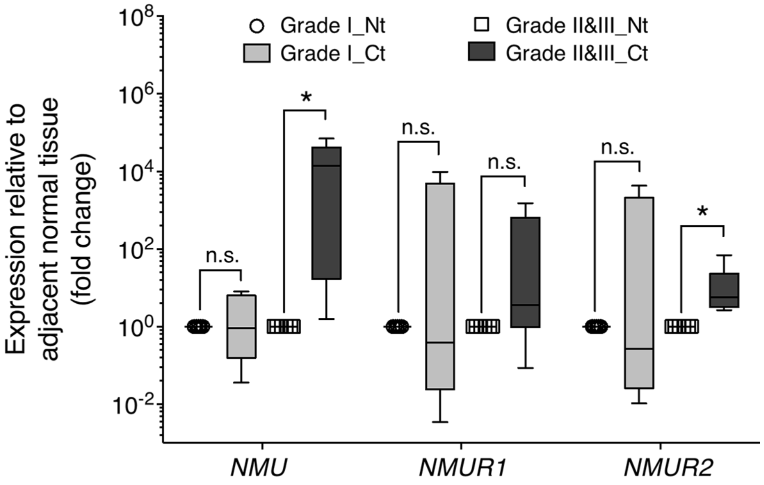


**Supplementary Figure S3: The transcript profiles of *NMU*, *NMUR1* and *NMUR2* in the human endometrial cancer.** The changes in transcript level of *NMU, NMUR1* and *NMUR2* in clinical specimens from patients with endometrial cancer, which have been divided in to the grade I ($n$ = 5) and the grade-II & III ($n$ = 6) groups. The gene transcripts were first quantified by real-time PCR and normalized against the corresponding *ACTB* level for each sample. Fold changes in each gene in the cancer tissue parts (*Ct*) were converted using the expression level in each corresponding adjacent normal tissue parts (*Nt*) as the one-fold control. The boxes represent the interquartile range; the horizontal lines in the boxes represent the median; the whiskers represent the minimum and maximum. n.s., no significance. *$p < 0.05$.

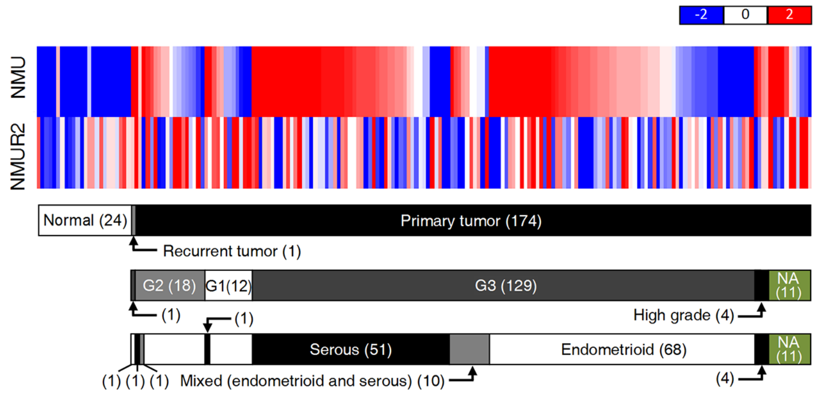


**Supplementary Figure S4: The patient characteristics and corresponding expression profiles of *NMU* and *NMUR2* in the cohort of TCGA uterine corpus endometrioid carcinoma.** The RNA sequencing dataset from TCGA was normalized across all cohorts in TCGA. The values of *NMU* and *NMUR2* extracted from the dataset were further $log_2$ transformed, normalized by the mean of transformed values and illustrated with UCSC Cancer Browser. Red, upregulation. Blue, downregulation. The patients' characteristics, including the sample types, grades and types of tumors, in the cohort of 199 cases were shown as bars below. The color in the bars represents the same category. The number in the brackets indicates the corresponding patient numbers. NA, information not available.

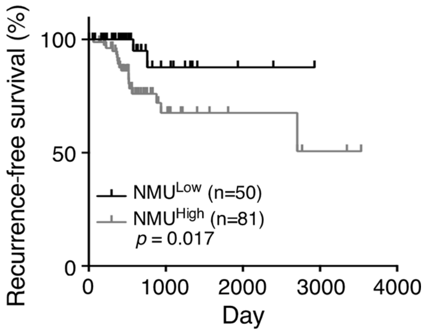


**Supplementary Figure S5: The relationship between the *NMU* transcript levels and recurrence-free survival in patients with endometrial cancer.** Patient data from the TCGA RNA sequencing dataset processed by UCSC Cancer Genomics Browser were divided into $NMU^{low}$ and $NMU^{high}$ groups according to the mean of $log_2$ transformed *NMU* values. The follow-up recurrence-free survival curves were then compared between these two groups.

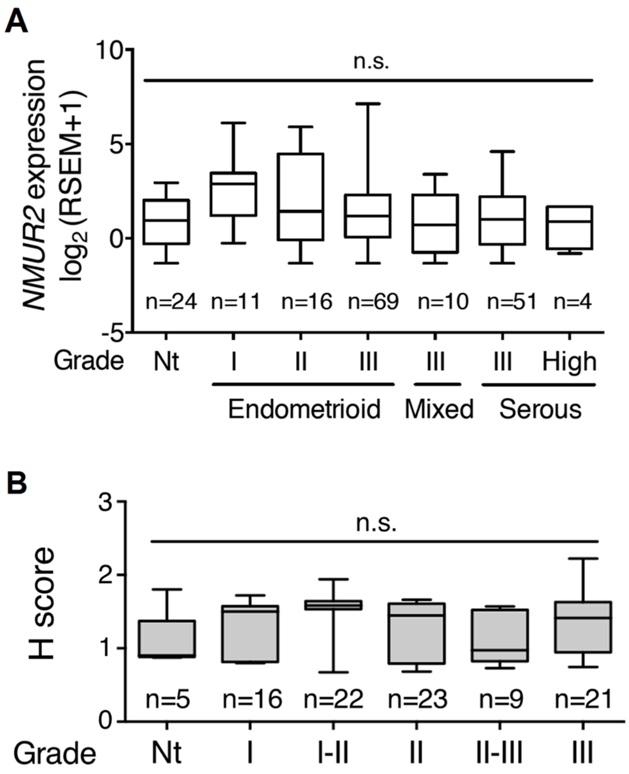


**Supplementary Figure S6: The expression profile of NMUR2 in the human endometrial cancer samples.** (**A**) The values of *NMUR2* extracted from the TCGA RNA sequencing dataset were $\log_2$ transformed by UCSC Cancer Genomics Browser and compared among normal tissues (Nt) and different grades and types of endometrial cancer. RSEM, RNA-Seq by Expectation Maximization. (**B**) Immunohistochemical analysis of NMUR2 in the endometrial cancer tissue microarray. The signal intensities of NMUR2 in the different grades of endometrial cancer were analyzed by the calculation of their H score. For *A* and *B*, the boxes represent the interquartile range; the horizontal lines in the boxes represent the median; the whiskers represent the minimum and maximum. n.s., no significance.

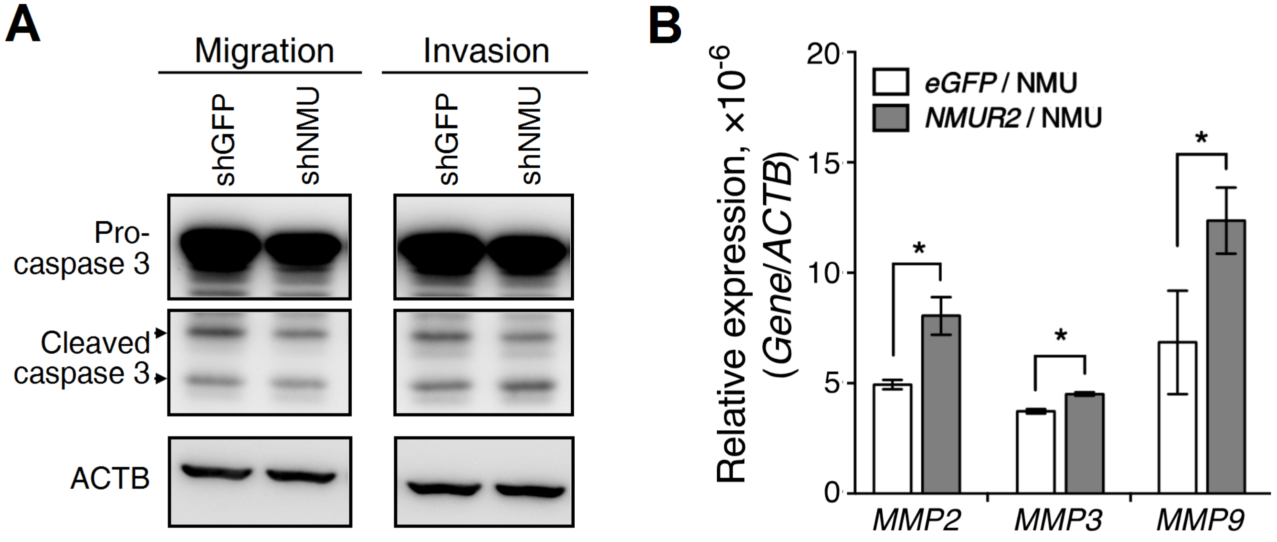


**Supplementary Figure S7: The effects of NMU signaling on the cell death and metalloprotease expression during the cell motility assays.** (**A**) GFP-knockdown control or *NMU*-knockdown RL95-2 cells were resuspended by serum-free medium and cultured respectively in the conditions used for the migration and invasion assays for 24 hours. The protein levels of pro-caspase 3 and cleaved caspase 3 were determined by Western blotting. (**B**) HEC1A cells overexpressing eGFP or *NMUR2* were cultured in the condition used for the invasion assay. Cells were treated with 100 nM NMU for 24 hours and the transcript levels of *MMP2, MMP3* and *MMP9* were further quantified by real-time PCR. Data were normalized against *ACTB* and are shown as the mean ± SD. *$p < 0.05$.

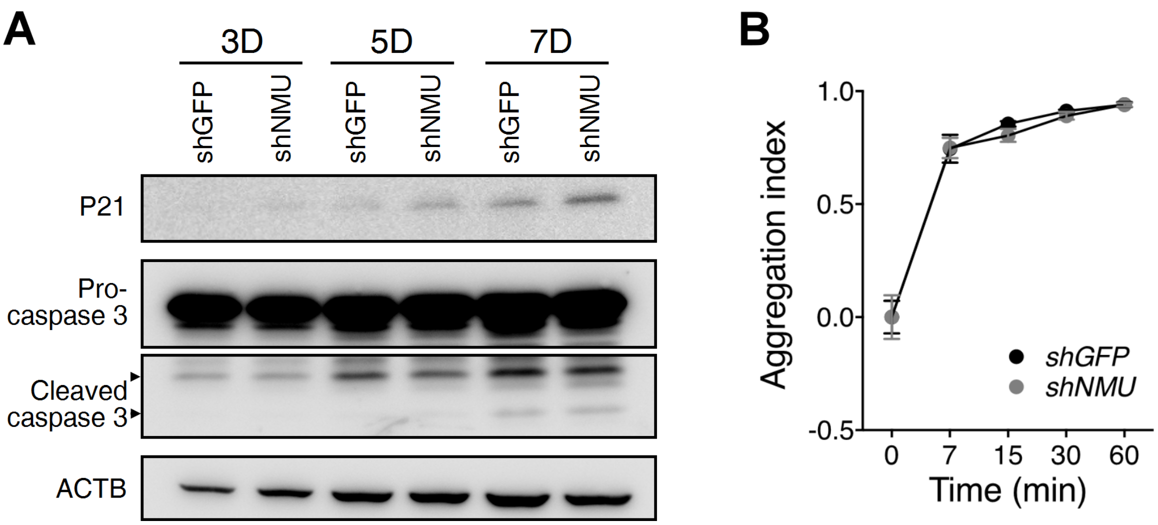


**Supplementary Figure S8: Effects of *NMU* knockdown in RL95-2 cells on the cell cycle, apoptosis and cell-cell aggregation.** (**A**) GFP-knockdown control and *NMU*-knockdown RL95-2 cells were cultured in the growth medium. The cell lysates harvested at indicated intervals were used to detect the protein levels of P21, pro-caspase 3 and cleaved caspase 3 by Western blotting. (**B**) GFP-knockdown control and *NMU*-knockdown RL95-2 cells were subjected to the cell-cell interaction assay. The converted aggregation indexes were compared at different intervals. Data are shown as the mean ± SD.

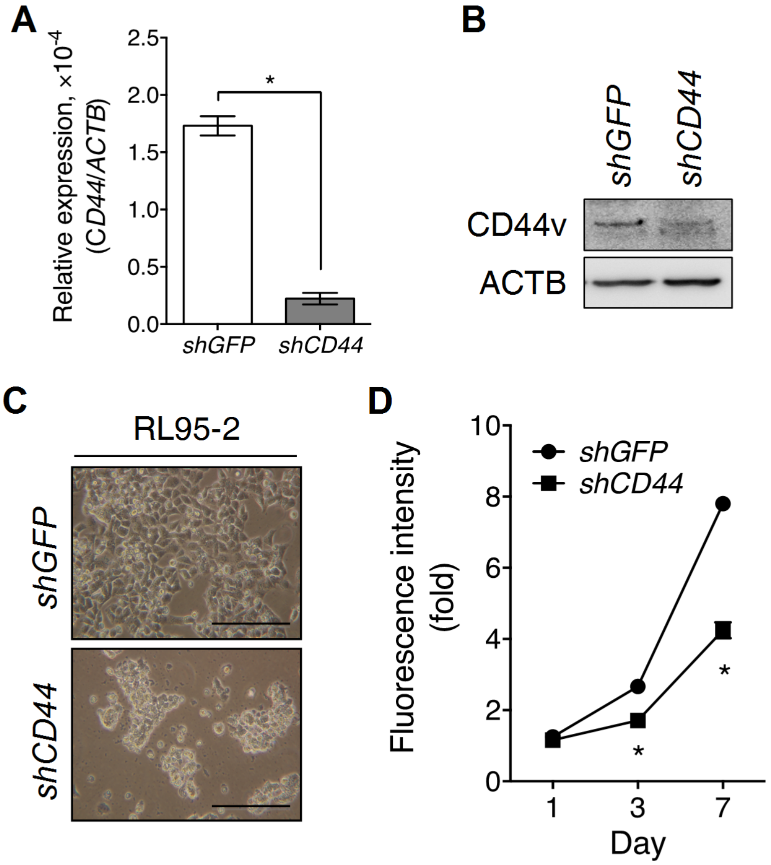


**Supplementary Figure S9: Effects of CD44 knockdown on the cell growth and morphology of RL95-2.** (**A**) The transcript level of *CD44* and (**B**) the protein level of CD44v were evaluated in *CD44*-knockdown RL95-2 cells. The transcript data are shown as the mean ± SD. *$p < 0.05$. (**C**) The morphologies of control and *CD44*-knockdown RL95-2 were observed on Day 5 of culture. Scale bar, 100 μm. (**D**) The growth rates were compared using the AlamarBlue assay. The fluorescence value of the cells on day 0 (D0) served as the one-fold control. Data are shown as the mean ± SD. *$p < 0.05$.

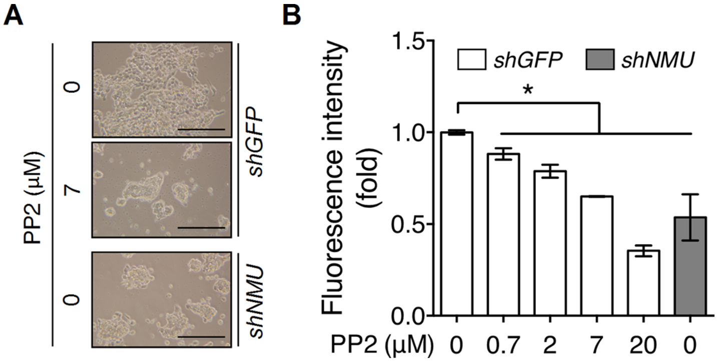


**Supplementary Figure S10: Inhibition of SRC shows phenotypes similar to knockdown of *NMU* in RL95-2 cells.** Control RL95-2 cells without or with PP2 treatment or *NMU*-knockdown RL95-2 cells were seeded onto the standard dishes for cultures. (**A**) The morphologies of the various cells were observed on Day 5 of culture. Scale bar, 100 μm. (**B**) The amounts of viable cells were measured by AlamarBlue on Day 7 of culture. The fluorescence value in control cells without PP2 treatment served as the one-fold control. Data are shown as the mean ± SD. *$p < 0.05$.

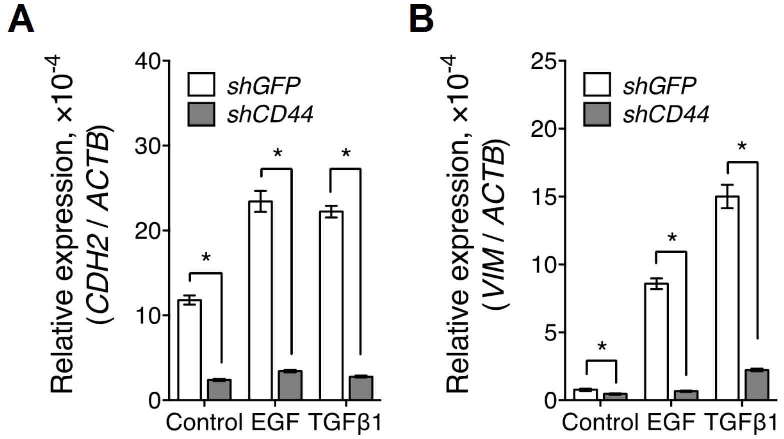


**Supplementary Figure S11: Knockdown of *CD44* attenuates the EGF- or TGFβ1-mediated mesenchymal marker expression in RL95-2.** The transcript levels of (**A**) *CDH2* (N-cadherin) and (**B**) *VIM* (vimentin) were compared between control and *CD44*-knockdown RL95-2 cells in the absence or presence of EGF (10 ng/ml) or TGFβ1 (3 ng/ml) treatment. The transcript levels from real-time PCR were normalized against *ACTB* and are shown as the mean ± SD. *$p < 0.05$.
